# How effective were government food box schemes for those who were shielding during the first wave of the COVID-19 pandemic in the United Kingdom? Local and national stakeholder perspectives

**DOI:** 10.1017/S1368980023001829

**Published:** 2023-12

**Authors:** Hannah Lambie-Mumford, Rachel Loopstra, Katy Gordon

**Affiliations:** 1 Department of Politics and International Relations, The University of Sheffield, Western Bank, Sheffield S10 2TN, UK; 2 University of Liverpool, Waterhouse Building, Block B, Brownlow Street, Liverpool, L69 3GF, UK

**Keywords:** Household food insecurity, Government intervention, Food parcel, COVID-19, Nutrition

## Abstract

**Objective:**

In spring 2020, governments across the UK put in place food box schemes to protect access to food for the population told to ‘shield’ from COVID-19 (i.e. not leave their house for any reason). This article explores the design, implementation and impact of food box schemes intended to regularly provide a week’s worth of food for individuals who were shielding.

**Design:**

Interviews and workshops with national and local stakeholders over summer 2020 to autumn 2021.

**Setting:**

England, Northern Ireland, Scotland and Wales.

**Participants:**

National and local government and NGO stakeholders involved in food response during the COVID-19 pandemic.

**Results:**

Local authorities played a crucial role, implementing and supplementing the national provision of food box schemes. Three key shortcomings of the schemes were identified: coverage, contents and accessibility. In England and Wales, the scheme only provided food for shielding individuals, not their household members. Across the schemes, box contents were criticised for not containing sufficient amounts of fresh or healthy food and for not being able to meet individual dietary requirements. They were also inaccessible for people who required support with lifting or preparing food.

**Conclusions:**

The inadequacy of shielding food box schemes may have undermined people’s ability to shield during the first UK lockdown. The COVID-19 pandemic required rapidly implemented policy responses, but these findings underscore the importance of universal provision and nutrition, physical accessibility and cultural food needs when formulating public health nutrition interventions.

A key part of the UK government pandemic response was to reduce exposure to COVID-19 among the population who were most at risk of serious illness from the virus. The policy of ‘shielding’ stipulated that people in this ‘extremely high risk’ group did not leave their home for any reason, including to shop for food^(1)^. For people in this group who were unable to get food delivered by other means (i.e. from friends, family, neighbours or supermarket delivery), governments in each UK nation established a grocery box scheme in the weeks following the issuing of shielding guidance. This paper analyses how the grocery box schemes were designed and implemented. It draws on data from local and national documentary sources and stakeholders collected as part of a project funded by the UK Research and Innovation Economic and Social Research Council COVID-19 rapid response grant scheme, that mapped and monitored responses to household food insecurity during the COVID-19 pandemic (March 2020 – January 2023).

On 22 March 2020, the UK government advised that people identified as at higher risk of severe illness from COVID-19 (‘clinically extremely vulnerable’) not leave their homes for any reason. This group was advised to ‘shield’ themselves from the virus and explicitly told to ‘not go out for shopping, leisure or travel, and, when arranging food or medication deliveries, these should be left at the door to minimise contact’^(1)^. The shielding guidance initially affected over 2·6 million people across the UK (2·2 million in England, 130 000 in Wales, 179 728 in Scotland, 90 000–95 000 in Northern Ireland)^(2–5)^. The guidelines were for an initial period of 12 weeks, but this was later extended to the end of July in England, Scotland and Northern Ireland, and 16 August in Wales^(2,6)^. The shielding guidance only applied to individuals with specific health conditions, not to other members of their household. The shielding guidance also did not apply to groups deemed to be at ‘moderate’ risk of severe illness from COVID-19 which covered a wide range of health conditions, including diabetes, asthma and chronic obstructive pulmonary disease, as well as people 70 years of age and older and pregnant women, though these groups were strongly advised to avoid going out as much as possible^(7)^.

Shielding guidance therefore limited physical access to food to doorstep food deliveries for the clinically extremely vulnerable population who lived alone or in a household comprised of shielding individuals, but also in cases where household members were not going out. Delivery options included supermarket delivery or from friends, families, neighbours or volunteers shopping on their behalf and delivering to the doorstep. However, several compounding factors compromised access to food through these means. Demand for supermarket delivery slots was extremely high in the early weeks of the pandemic and changing shopping patterns across the population at that time (who were all asked to only go out when absolutely necessary and as infrequently as possible) impacted on the availability of food in shops, in some cases prompting retailers to limit the amount of food that could be purchased by customers or the times that different groups of customers could enter shops^(8)^. Restrictions were also placed on out of local area travel, which meant friends or family of shielding individuals may not have been able to travel to different areas to provide support. With the closure of the hospitality sector (including cafes, restaurants and initially takeaways) and community venues (including day and community centres), access to food was further limited.

In light of these challenges, governments across the UK implemented interventions in an effort to ensure food access for the shielding group. Interventions in all four nations (England, Northern Ireland, Scotland and Wales) included both the grocery box schemes and facilitating priority access for supermarket delivery slots. Priority access schemes involved governments passing details of people on the shielding list onto retailers so they could be given priority for delivery slots. Major supermarket chains, namely Asda, Tesco, Sainsbury’s and Iceland, were part of these schemes across the four countries, and Morrisons, Waitrose, M&S, Co-op and Ocado were also involved in different country schemes^(6)^.

The Department for the Environment, Food and Rural Affairs (Defra) in England, the Northern Ireland Executive, Scottish Government and Welsh Government all established grocery box schemes between 29 March and 6 April 2020, intended as an option for those shielding who could not get food delivered to their doorstep by any other means. The boxes were intended to provide a week’s worth of primarily ambient food that could be stored at room temperature. Two of the UK’s largest food wholesalers, Bidfood and Brakes, were procured to deliver the schemes in England, Scotland and Wales, and it was announced that these companies would also oversee the delivery of standardised boxes to people’s homes^(9)^. In Northern Ireland, the scheme was run through the Department for Communities working with councils and voluntary and community organisations, as well as private firms^(10)^. People on the shielding list registered for boxes either through a telephone hotline or website (England and Northern Ireland), via a government SMS service (Scotland) or by contacting their local authority (Wales; people could also contact their local authority to receive a box in Scotland). In England, Wales and Scotland, people had to be on their respective government’s shielding list to be eligible for a grocery box and had to declare that they could not source food by any other means.^1^

The schemes ended with the shielding policy at the end of July 2020 (and mid-August in Wales). In Scotland, of an approximate number of individuals on the shielding list ranging between 170 000 and 180 000 over the time of the shielding policy, data suggest around 50 000 to 70 000 grocery boxes were requested each week^(11)^. In Wales, around 130 000 persons were on the shielded list by July 2020^(12)^, and an average of 11 300 food parcels were being delivered each week. As of August 2020, the scheme had delivered 214 711 food parcels to people who were shielding^(13)^. No final data for England or Northern Ireland were found up to the pausing of shielding at the end of July, but earlier reports provide some insight into the scale: 150 000 boxes had been delivered in Northern Ireland by the middle of June (around 80 000 people were shielding), and as of late June 2020, 300 000 boxes were being delivered each week in England (around 2·2 million people were shielding)^(14,15)^.

In light of the severe shielding restrictions placed on those who were clinically extremely vulnerable to COVID-19, there is a critical need to examine how, and how well, policy responses met the basic needs of this vulnerable group. There is a growing international evidence base on various pandemic food assistance responses targeted at different population groups over the course of the COVID-19 pandemic. Support to low-income families with children through school food replacements during school closures and non-governmental emergency food assistance are both a focus of existing international literature^(16–20)^. Common across this research is an emphasis on the importance of a range of stakeholders in the practice and implementation of support structures throughout the crisis. To our knowledge, this is the first study internationally of a government-provided food parcel intervention not targeted at a school-based population during COVID-19. Drawing on data from national and local policymakers and practitioners, this paper examines the design, implementation and impact of the national grocery box schemes across England, Northern Ireland, Scotland and Wales to evaluate its effectiveness in relation to food provision and its ability to protect this extremely vulnerable group from the virus. The paper addresses four research questions. First, how were the shielding box schemes designed? Second, how was provision implemented and what were the experiences of this from the perspective of different stakeholders? Third, what was the reach and impact of this intervention? And, lastly, how did provision and experiences differ between UK nations?

## Methods

**Fig. 1:**
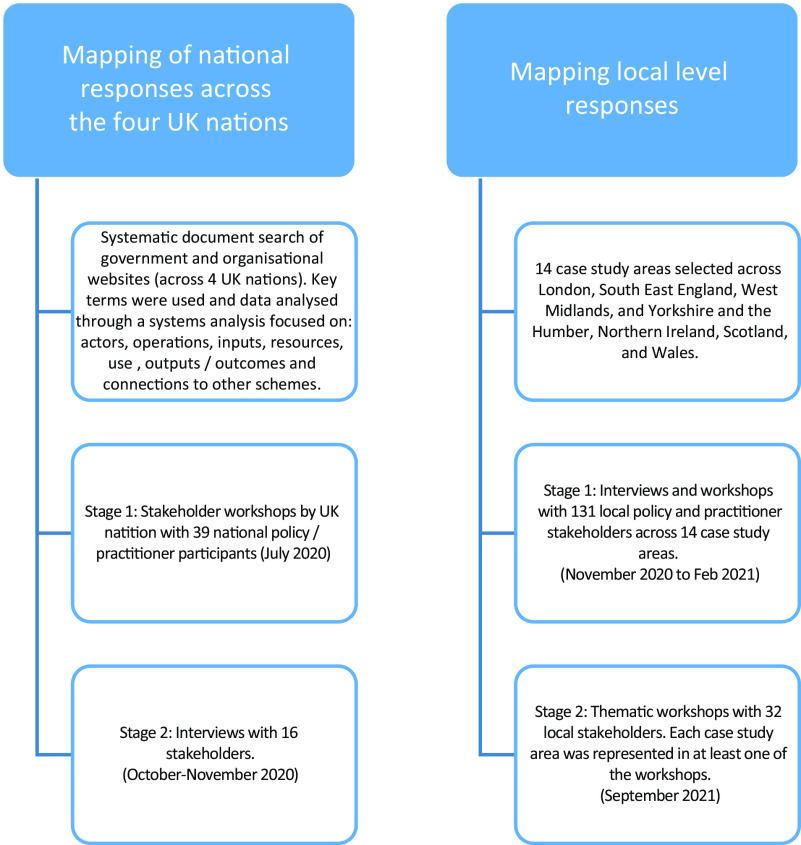
Methodology overview

The research was part of an 18-month mixed-methods project to map and monitor responses to risks of rising food insecurity in the UK during the COVID-19 pandemic (see Fig. 1). The project was funded through the UK Research and Innovation Economic and Social Research Council COVID-19 rapid response grant scheme. The research methods received ethical approval from the lead academic institution.

This paper draws specifically on qualitative stakeholder data relating to the shielding grocery box scheme, collected through two methods. First, in the summer of 2020, a series of online workshops were held with thirty-nine national policy and practitioner stakeholders as part of wider work to map and monitor national responses to concerns about food access under the COVID-19 lockdown. These workshops were followed by a short round of purposively sampled stakeholder input, including one-to-one interviews with sixteen stakeholders in autumn 2020 to fill specific knowledge gaps.

The second part of the methodology comprised fourteen local area case studies, with two waves of data collection (in winter 2020–21 and September 2021) involving local stakeholder interviews and online workshops. Interviews and workshops were conducted with a total of 131 local policy and practitioner stakeholders across the fourteen areas for the first wave of data collection; for the smaller-scale follow-up work, thirty-two stakeholders took part in thematic workshops (which explored the work of local authorities, food partnerships and charitable food aid). Six case study areas were in England, four in Scotland, two in Northern Ireland and two in Wales. In England, areas covered included London (two cases), South East England, West Midlands, Yorkshire and the Humber (two cases). In Northern Ireland, the cases were located in County Antrim and across counties Londonderry and Tyrone. In Scotland, cases were from Aberdeen and North East, Edinburgh and Lothians, Glasgow and Strathclyde, and the Highlands and Islands. Case studies in Wales were in the South East and South West of Wales. To maximise comparisons between responses serving rural and urban populations, the case studies were selected to include places that were either predominantly urban or predominantly rural and with evidence of the pandemic having an economic impact on the population (as reflected by rising benefit claimant rates). Among these, four were selected because they had local Food Power network alliances working in them, to allow us to explore the impact of these networks in the wider study. Detailed methodological appendices and reports have been published relating to both aspects of the research^(6,21–23)^.

### Analysis

The stakeholder data referred to above covered all responses to household food insecurity in the UK during the pandemic response. For the analysis presented in this paper, we first identified all data relating to the grocery box scheme and collated this into a new derived dataset. We then designed an analytical framework to answer our research questions, focused on key themes of box contents, the role of different actors in provision delivery, scheme coverage and accessibility.

## Results

### Design and implementation of the scheme

National policy stakeholders in Scotland reflected on the numerous issues that arose urgently and simultaneously when they were designing government support for people on the shielding list, including not only the grocery box scheme but also wider types of support that could be made available. These issues included the need for shielding, the unique circumstances imposed by the national UK-wide lockdown, the lack of online shopping delivery capacity, how to identify individuals in need of support, how to access food and how to deliver support in ways that met the need at pace. They also highlighted that the grocery scheme in Great Britain (England, Scotland and Wales) was novel:

‘During many years of planning and exercising for a Flu Pandemic, I never experienced any reference to a UKG/4-nation Shielding or a food box contingency plan. As far as I know, this was a novel contingency plan which was conceived, planned and deployed at incredible pace to save the lives of hundreds of thousands of our most vulnerable citizens.’ (National stakeholder, Scotland)

However, despite the pace that the national schemes were announced and rolled out (ranging from 1–2 weeks after shielding guidance was formally issued), local case study data highlighted that local authorities and other local actors had to support the shielding population in between the shielding policy being announced and the national box schemes being fully implemented. As highlighted in the key themes shown in Table 1, in England, one council shared with us that they provided food parcels to people on the NHS shielding list as a ‘stop-gap’ until the Government’s shielding food box programme was fully established. This was in response to concerns that the Government’s shielding boxes were not being signed up for fast enough, nor were they distributed fast enough or consistently in the first weeks. For these reasons, this local council-led provision ran from the end of March until the end of May 2020.

**Table 1:** Findings relating to the role of different actors involved in provision delivery

Local authority role in implementing the grocery box scheme	‘[Local authorities] were the key sectoral partner, in regards the design and delivery of Shielding here in Scotland. […]. We agreed that the standard Government provision, whilst meeting nutritional standards, would be considered as the core-element of provision, that may be adequate for some, but which would be supplemented via local authorities’ (National stakeholder, Scotland) ‘We knew of its shortcomings (variety) and strengths (pace, reach, scale), and worked with a huge range of partners to make sure that other parts of the system complemented the service.’ (National stakeholder, Scotland)
Local authority role in supplementing the grocery box scheme	‘Likewise, we found in the very beginning, for sort of the first two or three weeks, there was quite a deal of confusion among local authorities while everything settled down as to whether we were going to be supporting the shielded group or whether the local authority was. By about two, three weeks in, that had all cleared up.’ (National stakeholder, Scotland)

In our case study areas, we also heard how council staff had a role in reaching out to people on the shielding list to make sure they knew what they were entitled to and how to access support. For example, one of our case study areas in Wales shared that the council phoned everyone on the shielding list and if no one picked up after three times, they would visit their home. In both Wales and England, at the end of the shielding policy, we found examples of councils that called people on the shielding list to let them know that the food box programme was ending and to offer support in other ways, including access to priority supermarket delivery slots.

Our findings also show that there was a significant involvement from local governments in implementation and supplementation of these schemes, as can be seen in Table 1. In Northern Ireland, the intervention was designed so that last-mile delivery of food parcels to people’s homes was the responsibility of councils and local organisations. In Scotland, case study stakeholders highlighted that the local council provided fruit and vegetable parcels in addition to the Scottish Government’s shielding box. In case study areas in England, councils provided support to the shielding population to fill specific gaps in provision. In Wales, one council took over last-mile delivery following concerns over parcels going missing or being stolen, and in another, the council provided a food parcel scheme for people for whom the national box provision was inappropriate.

It is therefore clear from our local case study findings that local authorities played a crucial role in supporting the shielding population and implementing and supplementing the national grocery box schemes. Data from national stakeholders also supports this, and highlights how local authorities were seen as critical to the policy infrastructure providing support to – and improving outcomes for – this population:

‘As the contents of the box were limited – especially for particular dietary and cultural requirements – the Scottish Government did not see the food box as being sufficient for everyone’s needs in itself. […] It is important therefore, not to consider the impact of the grocery box on its own, but as part of a wider solution to food access which encompassed local authorities, voluntary and private sector support.’ (National stakeholder, Scotland)

However, as reflected in the following quotes, there were concerns about how well the different roles and responsibilities of local and national governments and other actors in supporting the shielding population were communicated, resourced or worked through in policy design and implementation:

‘Scottish Government’s view was, ‘Yes, we agree, if we’re providing food to shielding households as a government, it would be a good idea to provide fresh food’. Otherwise, it was all going to be ambient parcels and tins. ‘Yes, we agree that should happen. Over to you, local authorities, to make that happen’. We’re like, ‘Woah, what do you mean we’re now doing fresh food. Yippee, excellent’. (Local stakeholder, Scotland)

In one area in England, participants also reflected on challenges around the communication and data sharing required between national and local government to help support the shielding population:

‘What didn’t work well initially was some of the information that we were receiving from the Government in respect of who was clinically extremely vulnerable or, in the first phases of this, called shielding. Some of the data that was coming out that we needed to use to help our communities to know who would be getting a government food parcel, etc., some of that data flow was not good.’ (Local stakeholder, England)

### Shortcomings of the shielding grocery box schemes: coverage, contents and accessibility

The research findings highlight three key shortcomings of the national shielding grocery box schemes, as can be seen in Table 2.

**Table 2:** Findings relating to the shortcomings of the shielding grocery box scheme

Scheme coverage	
Support not provided to whole household (England and Wales)	‘The ones that came from Brakes [funded by the Welsh Government] were, literally, for a shielding person. There was not enough food in there for a week. I saw what was in one. There is no way that that was enough for even one person for a week, and if you’re shielding… They were shielding with their whole family’. (Wales local stakeholder)
Grocery box contents	
Lack of fresh or healthy food	‘We’ve also had reports that quite a lot of the fresh fruits or fresh vegetables have been a bit of an issue. […] They’re worried about the carrots because they’re almost not fit for purpose. [People have] been unable to eat [them] because they’re not fresh enough. They’ve just been black and liquid too quickly for people to be able to use.’ (National stakeholder, Scotland) ‘By the time we got involved, the contents of those boxes had been decided on, and going forward, it would certainly be nice to look at the nutritional value. Especially given that those shielding people, and the people who have limited access to food are likely to be more nutritionally vulnerable.’ (National stakeholder, Northern Ireland) ‘I think our food parcels were better than the shielding food parcels because we took on board the advice of our dietary colleagues in the NHS… we got them to analyse, after a few weeks, what was in our food parcels for those isolating who couldn’t afford food, to make sure it was nutritious and balanced.’ (Local stakeholder, Wales)
Not able to meet dietary requirements	‘…there was also a question around to what extent those food parcels met people’s dietary needs. […] But I think it was that they’d just got the one option, there wasn’t an opportunity to make the food boxes any more sort of bespoke.’ (National stakeholder, Wales) ‘With the letters that went out from Welsh government there was a lot of, ‘If you need more help speak to your local authority, here is a list’. Obviously that placed the onus on us to provide additional information. But as soon as we would see what was in the parcels and the fact that they were not covering an awful lot of scenarios for people, it was something that we tried to do, to help out where we could.’ (Local stakeholder, Wales) ‘If we were contacted on the hub, that the boxes weren’t culturally sensitive, for example, then we would work with our local food providers to see if we could change it in any way, but we didn’t receive that many comments about them being culturally insensitive or, indeed, not meeting the needs of people who had medical conditions. We received a few and we got round it.’ (Local stakeholder, England)
Accessibility	
Inappropriate for people who required support with lifting or preparing food	‘If people couldn’t physically pick up a box from their doorstep, [the] Council would provide the boxes for them instead so that social services could go in with an actual box and make sure that it wasn’t just left on the doorstep’. (Local stakeholder, Wales)

Firstly, in England and Wales the national schemes were targeted at the individual shielding and didn’t take account of the wider household composition. Only people on shielding lists were eligible for government grocery box support, meaning that others in their household would still have to leave the house to shop for food unless they could request food from other sources, such as food banks, as illustrated in the following quote:

‘[…] for a number of people who were entitled to a shield box, the reason they didn’t take it up, and instead [requested food from] the food bank, is because food bank parcels are there for the whole household, whereas shield boxes are only there, of course, for an individual within a household who is shielding.’ (National stakeholder, Wales)

The lack of coverage of the schemes for everyone in a shielding person’s household was seen to result in risking people on the shielding list being exposed to the virus, as household members were not enabled to shield alongside their vulnerable family member.

It is of note that this risk appeared to be mitigated in Scotland. Data on the scheme in Scotland, released following a freedom of information request, highlighted that people who were shielding were able to request more than one box ‘if they had dependents or others in their household, who were also not able to leave the house to buy groceries’ (Scottish Government 2020). Based on the freedom of information response, we calculated that about 13 000 to 21 000 additional boxes were delivered to the households of people shielding each month to meet the needs of their wider household. Those were in addition to the 43 700 to 50 800 delivered to meet the needs of individuals.

Secondly, the box contents were also seen as inadequate by many local and national stakeholders. Data from stakeholders highlighted that contents were not appropriate for recipients and did not contain sufficient fresh and healthy food:

‘Massive catering tins of fruits or vegetables or beans, or salad beans are some of the things that have been reported to me that an older person shielding couldn’t, in a million years, manage to eat.’ (National stakeholder, Scotland)

Concerns were raised over the nutrition quality and quantity, particularly the lack of fruit and vegetables, or consideration for dietary and cultural needs. The fact that dietary needs could not be catered for by the schemes was seen as a significant limitation:

‘[We had] quite a lot of queries come into the clinical dietetics service about those with special dietary requirements and where they were going to be met. So special diets and things like that, where the national food parcels were not meeting their needs, particularly from paediatrics as well…there were clinical needs that were arising because of [the lockdown].’ (Local stakeholder, Wales)

Whilst we collected evidence of councils stepping in to provide food support for those on the shielding list with dietary needs to overcome this limitation of the national schemes, they also talked about how challenging this was logistically:

‘We also tried to cater for dietary requirements, but that became very complicated, I’ve got to say. I’m not sure how successful that actually was. I think they were often vegetarian. I think they were often halal and stuff like that, but I feel that’s as good as you got.’ (Local stakeholder, Wales)

Thirdly, there were also issues of accessibility. Confusion over who was entitled to support was highlighted in local case study data, relating to difficulties accessing the lists of people who were in the shielding group.

‘The government department didn’t hold that list, and it was all done through GPs which created another level of complexity and slowed things down further.’ (Local stakeholder, Northern Ireland)

Other questions were raised about the appropriateness of the provision design. As already highlighted, one council in Wales provided an alternative food box service to people who could not physically pick up the national food boxes from the doorstep. In Northern Ireland, one local stakeholder reflected that for some people in the shielding population, a food parcel was not a suitable replacement for the food support they might normally have received:

‘I did the COVID helpline for six weeks here, and what we realised was that the food boxes were not suitable for the client group that they were supposed to be for, because the client group that they were for, were isolated, older, vulnerable, disabled sick people who actually in many ways needed a hot meal because they were so used to family coming in and doing that for them, but the family were taking a step back, and therefore it was a complete miss-match of approach.’ (Local stakeholder, Northern Ireland)

## Discussion

Our findings show that local authorities played a crucial role in supporting the shielding population and implementing and supplementing the national grocery box schemes across the period shielding guidelines were in place (March – August 2020).

They also highlight three key shortcomings of the schemes. In England and Wales, the schemes did not provide food support to the whole household, only the shielding individual, potentially increasing the risk of exposure. Across the nations, the food box provision was seen as inadequate by many local and national stakeholders because it did not include fresh and healthy foods or meet specific dietary needs. The provision was also inaccessible for people who required support with lifting or preparing food.

Our findings are supported by other evidence, which highlights shortcomings around the targeting of the schemes, food box contents and accessibility^(24–26)^. For example, a survey of service users carried out as part of an evidence review by the Environment, Food and Rural Affairs committee found that only 44 % of those who had received a grocery box said that the contents had met their needs^(24)^. Insufficient quantities, the unsuitability of foods provided and a lack of nutritional balance were common reasons for this. A study by the Poverty Alliance in Scotland also highlighted concerns about the quality, adequacy and regularity of food provided in the grocery boxes^(25)^. In contrast, one nutritional analysis compared the contents of the standard food parcels to the recommended intakes of macro- and micronutrients for women aged 50–64 and found that the boxes met the nutritional needs of most adults^(27)^. Notably, however, people with special dietary needs were not accounted for in this analysis.

In relation to the accessibility of the support, despite the fact this provision was targeted specifically for people who were shielding, insights collected from community activists, reported by the Poverty Alliance in Scotland, suggested that many people who were shielding felt they were missing out on support due to inconsistencies in the level of support made available by local authorities and a belief that the criteria for shielded groups had not been well-communicated in some areas^(25)^. The British Red Cross noted that some people may not have been able to understand or action the ‘notes’ included in the boxes, for example, those explaining that people should contact their local authorities if the box wasn’t suitable, due to language barriers^(26)^. As in our case study findings, another concern was people not being able to lift the contents of the box into their house^(26)^.

Our findings are also consistent with international literature on other types of food interventions elsewhere during the COVID-19 pandemic. Common across this research is an emphasis on the importance of a range of stakeholders in the practice and implementation of food support structures throughout the crisis^(16–20)^. Evidence suggests that support was particularly successful where there were preexisting relationships and good communication^(16,17)^.

### Implications

Our findings suggest shortcomings in the national grocery box schemes for people who were shielding between March and August 2020 including coverage, contents and accessibility. In Table 3, we set out a number of recommendations for future research and public health planning. As the only intervention with the potential to ensure people who were shielding had their dietary needs met and that they did not have to leave their homes for food, it is significant that this intervention did not appear to deliver this protection. The national-level interventions did not have a mechanism in place to provide for special dietary or cultural needs, physical requirements or for household members of people shielding. Whilst it is clear from our findings that local authorities were expected to make up for these shortfalls in the national schemes, there was a lack of clarity about how local authorities were supposed to do this, and our findings suggested significant variability in their ability to do so and what was done in response. This ultimately must have led to inconsistent support for people who were shielding across the country.

**Table 3:** Recommendations for future research and public health planning

Recommendations for future research: key questions	From the perspective of people who received the grocery boxes: What were the challenges different people faced? How helpful was the provision to them? Were there particular needs around food preparation and eating support that were unmet? In a larger sample of areas, were there any differences in the implementation of the intervention according to deprivation indices? What are the roles and responsibilities of actors at different scales in support of food access during emergency responses? What should the role of national and local governments be? What are the key lessons stakeholders took from this provision? And how are they implementing those lessons into future crisis planning?
Recommendations for public health planning	Focus attention on ways to include fresh fruits and vegetables and rigorously plan for how this can be implemented in practice. Clear plans should be drawn up for how food support can be adapted for special diets, and how this can be done at scale. Public health emergency planning should include potentially at-risk groups in processes of emergency response co-design. Non-government partners should also be part of this co-design process. Emergency planning should also consider other kinds of food provision, particularly prepared meal provision for those with additional food support needs.

### Strengths and limitations of the research

This study has strengths but also limitations. First, the scale of participant recruitment was significant. For the national-level research, thirty-nine government and NGO participants with direct experience of the design and/or implementation of the shielding box schemes took part in the research. In the area case study research, 131 local stakeholders took part, representing a diverse mix of public and voluntary sector experience of the design and implementation of the schemes and surrounding support at a local level. Another strength is the ability to compare findings across the four nations of the UK and across local areas.

The key limitation of this research is that data were not available to fully evaluate the grocery box schemes. Accessing government data was extremely challenging, as was collecting data from participants involved in front-line delivery during a time of crisis. It would have been useful to know what proportion of people who needed grocery boxes received them and on what timescales, and for representative survey data to have been collected on their use and ability to meet dietary and cultural needs among people who received them. It would also have been useful to understand whether the experiences observed in our case study areas were common across local authorities. What our data do provide are comprehensive insights from national and local stakeholders into the process of design and implementation of the national grocery box schemes across England, Northern Ireland, Scotland and Wales. These data point to key dynamics relating to the role of local government and some key challenges posed by the intervention design in relation to the coverage, contents and accessibility of the schemes.

### Conclusion

The limitations of national grocery food box schemes could ultimately have undermined the ability of people who were clinically extremely vulnerable to COVID-19 to shield, as the scheme was intended to support people who had no other way of obtaining food. Our data suggest there needed to be a universally available mechanism in place to respond to different dietary and cultural needs; ignoring these in the design of this intervention was a significant shortcoming. The provision of healthy food, especially appropriate amounts of good quality fresh fruit and vegetables, is also a critical nutrition requirement and again should have been built into the design of the food box contents. Future design and implementation should focus on wider household needs, food box contents and the accessibility of the provision. The implementation of these schemes saw significant involvement from local actors; however, localised responses can be variable; further research is needed to understand whether provision of this nature is best organised at a local or national level, and if at the local level, how equitable provision can be ensured. Whilst the COVID-19 pandemic required unprecedented policy responses, these findings highlight how important it is for public health policymakers to think through, as part of their emergency planning, how different emergency/crisis scenarios may impact on food access in the future for different groups, and how to provide universal access to responsive, appropriate and effective support.

## Footnotes

1
Eligibility criteria in Northern Ireland were broader: boxes were available through the government scheme for both people shielding and those not shielding but in critical need of food due to, for example, being disabled, isolated or having too little money.
